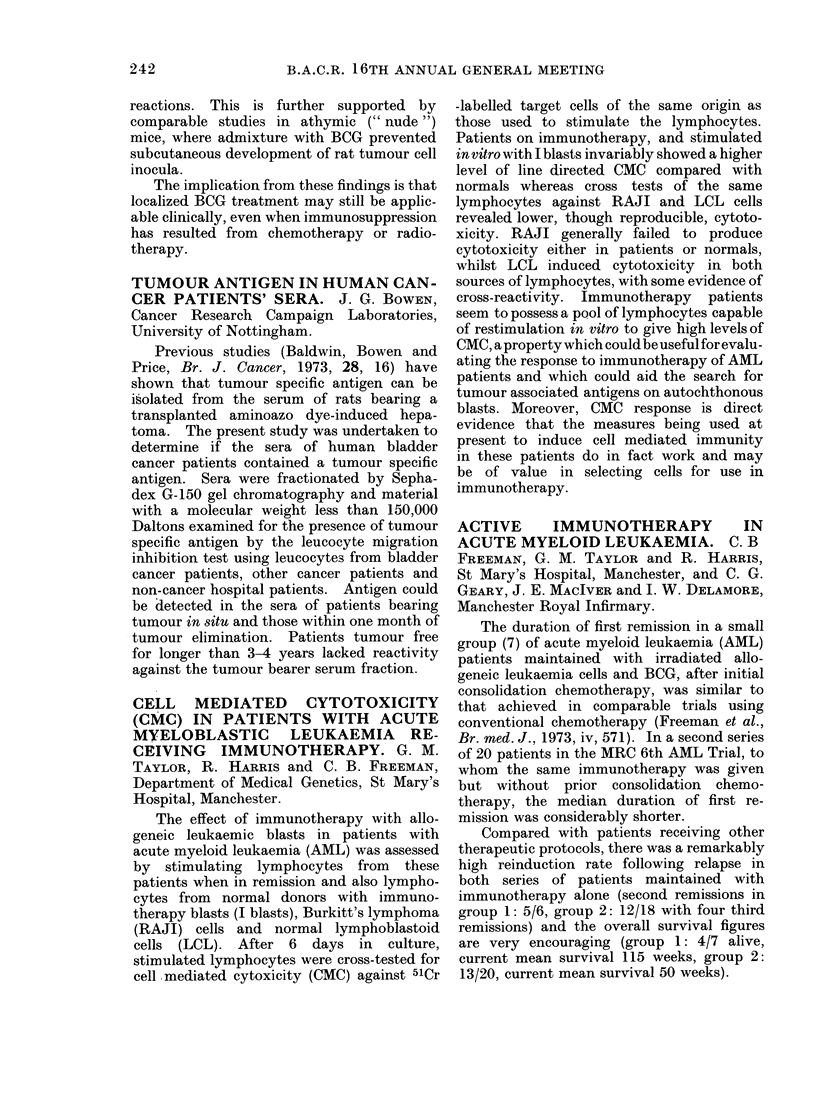# Proceedings: Cell mediated cytotoxicity (CMC) in patients with acute myeloblastic leukaemia receiving immunotherapy.

**DOI:** 10.1038/bjc.1975.162

**Published:** 1975-08

**Authors:** G. M. Taylor, R. Harris, C. B. Freeman


**CELL MEDIATED CYTOTOXICITY (CMC) IN PATIENTS WITH ACUTE MYELOBLASTIC LEUKAEMIA RECEIVING IMMUNOTHERAPY.** G. M. TAYLOR, R. HARRIS and C. B. FREEMAN, Department of Medical Genetics, St Mary's Hospital, Manchester.

The effect of immunotherapy with allogeneic leukaemic blasts in patients with acute myeloid leukaemia (AML) was assessed by stimulating lymphocytes from these patients when in remission and also lymphocytes from normal donors with immunotherapy blasts (I blasts), Burkitt's lymphoma (RAJI) cells and normal lymphoblastoid cells (LCL). After 6 days in culture, stimulated lymphocytes were cross-tested for cell mediated cytoxicity (CMC) against $^{51}$Cr-labelled target cells of the same origin as those used to stimulate the lymphocytes. Patients on immunotherapy, and stimulated *in vitro* with I blasts invariably showed a higher level of line directed CMC compared with normals whereas cross tests of the same lymphocytes against RAJI and LCL cells revealed lower, though reproducible, cytotoxicity. RAJI generally failed to produce cytotoxicity either in patients or normals, whilst LCL induced cytotoxicity in both sources of lymphocytes, with some evidence of cross-reactivity. Immunotherapy patients seem to possess a pool of lymphocytes capable of restimulation *in vitro* to give high levels of CMC, a property which could be useful for evaluating the response to immunotherapy of AML patients and which could aid the search for tumour associated antigens on autochthonous blasts. Moreover, CMC response is direct evidence that the measures being used at present to induce cell mediated immunity in these patients do in fact work and may be of value in selecting cells for use in immunotherapy.